# Preparation for fatherhood: A survey of men’s preconception health knowledge and behaviour in England

**DOI:** 10.1371/journal.pone.0213897

**Published:** 2019-03-20

**Authors:** Jill Shawe, Dilisha Patel, Mark Joy, Beth Howden, Geraldine Barrett, Judith Stephenson

**Affiliations:** 1 Institute of Health & Community University of Plymouth, Plymouth, United Kingdom; 2 Institute for Women’s Health, University College London, London, United Kingdom; 3 School of Health Sciences University of Surrey, Guildford, United Kingdom; 4 Bristol Medical School, University of Bristol, Bristol, United Kingdom; Aga Khan University, KENYA

## Abstract

**Methods:**

A cross-sectional survey of men attending antenatal care with their partners at three London Maternity Units. We assessed level of pregnancy planning using the partner version of the London Measure of Unplanned Pregnancy (LMUP), preconception health behaviours, and whether they had sought information and health professional advice before conception.

**Main results:**

We recruited 573 men (91% response rate). Mean age was 34 years, 86% were in employment or full time education and 66% had a degree. Half were overweight or obese, 16% were still smoking and 79% had consumed alcohol in the three months before conception. Of 250 men answering questions about medication, a third were taking medication with potentially adverse effects on male reproductive health, while 23% reported taking pre-pregnancy vitamins.

46.9% had looked at information about pregnancy from a variety of sources, including online, before their partner became pregnant. Assessed by the LMUP, 74% of pregnancies were planned. Male ‘planners’ were more likely than other men to reduce smoking, reduce alcohol consumption and to eat more healthily in preparation for pregnancy. However, 57% took no action to improve their health.

**Significance of the findings:**

In a sample of relatively educated men accompanying their partners on an antenatal visit, nearly half had made at least one positive health behaviour change before pregnancy, but half were overweight or obese and a third were on medication that could impair male reproductive health. These findings, together with a high prevalence of alcohol consumption and smoking, indicate the need for greater paternal preconception health awareness and care. Innovative ways to promote positive messages about fatherhood, including medication review as part of preconception care, should be evaluated for impact on improving paternal reproductive health and pregnancy and neonatal outcomes.

## Introduction

A growing body of evidence suggests that men’s health at conception plays a significant role in pregnancy and neonatal outcomes through direct and genetic influence of spermatozoa quality [1–3] and via psycho-social determinants of health [4]. There is current concern that semen quality is decreasing [5] and men’s awareness of factors that influence fertility is poor [6, 7]. Men’s reproductive health and specifically the quality and genetic integrity of spermatozoa is known to be affected by factors including advancing paternal age [8–12]; medical conditions such as obesity[13], diabetes[14, 15], hypertension[3, 16]; medication and recreational drug use [6] and lifestyle factors [7]. Recent research examining environmental and lifestyle exposure as an epigenetic influence on the paternal germline has shown the potential to influence health outcomes of future generations [3, 5–7, 17, 18]. Paternal influence reflects holistic bio-psycho-social factors across the lifespan that in addition to affecting sperm health, can also influence men’s own mental and physical health; their adaptation to fatherhood and the health of future generations for example through passive smoking and other health behaviours [4].

Preconception care (PCC) aims to enhance health status before conception in order to reduce perinatal morbidity and mortality and improve maternal and child health in the short and long term [19]. The World Health Organisation and Professional bodies have policy and guidelines covering biomedical, behavioural and social health interventions for preconception care [20, 21]. In the UK and in Europe interventions are mostly aimed at women [22] whereas in the USA improving men’s health is integral within the Centre for Disease Control (CDC) State preconception programmes [23, 24]. This policy recognises the role of men in family building and the benefit of including men in the preconception care agenda. However men are known to be poor users of preventative health services [25] and more likely than women to undertake risky health behaviours [26–28].

The period of spermatogenesis [29, 30] gives a unique opportunity for men to improve spermatozoa quality by modifying health behaviour before conception [5–7, 18]. This is reflected in Governmental policy recommendations for men to improve nutrition and lifestyle practices such as smoking and alcohol consumption before conception [22]. Other recommendations to promote preconception care include screening and treatment of sexually transmitted infection that may impact both on pregnancy and the partner; review of medications; screening and health promotion initiatives for intimate partner violence; and promoting deliberate decision making regarding fatherhood to ensure all pregnancies are intended [6, 31, 32].

There is little understanding of men and their preparation for pregnancy and our study sought to explore men’s knowledge and health behaviours prior to fatherhood through a questionnaire survey of men attending antenatal clinics with their partners.

## Materials and methods

### Study design

This study formed part of a larger project exploring Pre-Pregnancy Health and Care in England 006/0068 which gained favourable ethical opinion from NRES London-Bromley REC reference 11/LO/0881.

The findings from the pregnant women, which include a detailed methodology of this research and sample size considerations have previously been published [33].

We undertook a cross-sectional questionnaire survey of male partners of pregnant women, who were attending for an antenatal scan or booking appointment at three London hospitals. The hospitals were chosen specifically to provide participants from a wide range of ethnic and socioeconomic backgrounds and men were recruited through convenience sampling.

Men were approached by trained researchers whilst waiting for their appointment and in accordance with ethical principles, offered an information leaflet describing the study aims, objectives and consent process. Consent to participate was implied by virtue of completion of a pen-and-paper questionnaire returned to the researcher. The questionnaires were anonymous to ensure confidentiality and all data was held in accordance with the Data Protection Act 1998.

### Questionnaire instrument

The questionnaire was developed following a literature review exploring topics for inclusion and consideration of preconception questionnaires used in Sweden [34] and in the Southampton Women’s Survey [35]. It was then piloted with a diverse reproductive health user participation group formed from service users and members of the local community. The questionnaire followed a similar format to the women’s questionnaire [33] but was tailored to partners. It contained 54 questions mainly with a multiple-choice format, some providing the opportunity for additional free text.

Section one “Before your partner became pregnant”, asked whether any sources of pre pregnancy information and advice had been accessed and whether any health professionals had been visited for advice on becoming pregnant. Information regarding specific preconception health behaviours including being a healthy weight, eating a healthy diet, alcohol use, smoking, recreational drugs, sexually transmitted infections (STIs) and caffeine use were examined.

Questions inquired whether the men had taken specific vitamin and minerals as supplements before conception and to specifically list any taken; whether any body building supplements, sports nutrition and steroids had been used and to list what type; whether their partner had taken folate supplements and whether in the six months before the pregnancy they had been screened for STIs such as chlamydia.

The second section, “Now your partner is pregnant” asked about information sought and actions taken once pregnancy had occurred. Questions were included that explored pregnancy intention from the partner’s perspective. The version of the London Measure of Unplanned Pregnancy (LMUP) [36] adapted for partners was used to assess level of pregnancy planning. Topics covered by the LMUP items included contraception use, timing of fatherhood, intention, desire for a baby, partner discussion and agreement, and actions taken to prepare for pregnancy. Scores of 0 to 3 were categorised as ‘unplanned,’ 4 to 9 as ‘ambivalent’ and 10 to 12 as ‘planned’ [48][37].

The last two sections “About your Health” and “About you” collected information about health status including weight and height; any longstanding illness or disability; free-text listing of medications and the condition they were treating and completion of established socio-demographic characteristics.

### Statistical analysis

A professional data entry company (Abacus) input survey results into a database. Data were cleaned and verified by study researchers (BH & DP). SPSS was used for descriptive analysis and Chi- squared test for comparison of categorical variables: age, education, employment, general health, BMI and level of pregnancy planning.

Further analysis was undertaken using R version 3.2.4 utilising library routines from mice package 2.25 (MJ). We used predictive mean matching, imputing ten data sets. We assumed that the data were missing at random (Little test for MCAR, p<0.003). The variables used in imputation were Age Group, Ethnic Group, General Health, Academic Qualification, Employment Status and LMUP score. No statistical interactions were included. Logistical regression was performed for unadjusted and adjusted odds ratio in relation to behaviour change with 95% CI.

## Results

Overall, 622 men were invited to participate in the study and 573 completed the survey, giving a 91% response rate. 49 declined for various reasons (did not want to take part, n = 16; language barriers, n = 15, too busy n = 13; feeling unwell, n = 3 and no reason, n = 2).

Table 1 shows demographic characteristics and level of pregnancy intention using the LMUP for all participants.

**Table 1:** Demographic characteristics and level of pregnancy intention.

Characteristic	% (n)
Total	n = 573
**Age**	
**< = 25**	5.2 (26)
**26–30**	19.2 (97)
**31–35**	39.7 (200)
**36–40**	23.6 (119)
**41–45**	9.3 (47)
**> = 46**	3.0 (15)
**Missing**	(69)
**Ethnic group**	
**White**	73.7 (382)
**Mixed**	4.4 (23)
**Asian**	12.3 (63)
**Black**	6.6 (34)
**Chinese or Other**	3.1 (16)
**Missing**	9.5 (55)
**General Health**	
**Excellent**	30.4(161)
**Good**	59 (312)
**Fair**	10.6 (56)
**Poor**	0
**Missing**	7.7 (44)
**BMI Group**	
**Normal**	48.9 (246)
**Overweight**	49.7 (250)
**Underweight**	1.4(7)
**Missing**	(70)
**Employment Status**	
**Employed or full-time education**	86.5 (448)
**Part-time**	6.9 (36)
**Unemployed**	4.1 (21)
**Other**	2.5 (13)
**Missing**	(55)
**Highest Academic Qualification**	
**Degree**	66.7 (338)
**Diploma**	10.8 (55)
**A/AS/S levels**	5.5 (28)
**Other**	17 (86)
**Missing**	(66)
**Pregnancy Intention** **LMUP Score**	
**0–3 Unplanned**	1.2% (7)
**4–9 Ambivalent**	24.9% (140)
**10–12 Planned**	73.9% (416)
**Missing**	(10)
**First Time Father**	
**Yes**	66.5%(344)
**No**	33.5%(173)
**Missing**	(56)

The mean age of the male partners who completed questionnaires was 34.1 years (range 18–52 years). 86.5% (n = 448) were employed with 66.7% (n = 338) having a degree. Of those answering the question regarding whether they already had children (n = 517) 66.5% were first time fathers. The majority of men completing the LMUP had scores suggesting pregnancy was planned (73.9% n = 416). 7.9% (n = 45) stated that they had undergone fertility treatment for the current pregnancy.

A majority of men rated their health status to be good or excellent (84.1% n = 482). 49.7% of men in our sample had a Body Mass Index (BMI) of over 25, 1.4% (n = 7) were underweight with a BMI less than 18.5 and 48.9% had a normal BMI between 18.5 and 24.99.

7.5% (n = 43) stated they had a long standing illness. Specific health conditions mentioned included respiratory and asthma (21.9%), allergies (17.1%) gastric/ intestinal (13.5%), dermatological (11.4%), cardiac (8.6%), mental health (6.7%), diabetes (2.9%), epilepsy (1.9%), kidney (1.9%), and arthritis (1%).

Of 250 men who answered questions about medication, 32.8% (n = 82) were taking medications that had the potential for adverse effects on male reproductive health [38] for example anti-epileptic medication, anti-depressants and 5-alpha-reductase inhibitors[39–41]. Under half of these (48.8% n = 40) had had medications reviewed by a doctor in the three months before conceiving. 13.6% (n = 78) had been for an STI check before conceiving.

### Preconception information and advice

Almost half of men, 46.9% (n = 266) had looked at information from a variety of sources (including information online) about their partner becoming pregnant before conception.

More men had received advice before pregnancy from family/ friends (FF) or other sources as opposed to from General Practitioners (GPs) and Health professionals (HP) (Table 2). 45.7% (n = 262) stated they were given no advice or sought no advice for themselves.

**Table 2:** Information about preconception health behaviours given to the men before pregnancy by source. (Nb. participants were able to tick multiple sources).

Preconception Behaviours	GP	Other Health Professional	Family/Friends	Other*
Eating a healthy diet	6.8 (39)	2.8 (16)	19.3(111)	14.6(84)
Knowing if you were the right weight for your height	5.0 (29)	1.7 (10)	3.5(20)	8.0(46)
Taking vitamins supplements	5.2 (30)	1.7 (10)	8.0(46)	9.6(55)
Caffeine	4.5(26)	1.7 (10)	7.8(45)	9.2(53)
Alcohol	7.2(41)	2.4 (14)	14.7(84)	15.4(88)
Smoking	7.3(42)	1.9 (11)	12.7(73)	13.4(77)
Street drugs	3.3(19)	1.7 (10)	7.7(44)	10.1(58)
Immunisations	2.8(16)	1.2 (7)	2.4(14)	3.3(19)
STI Info	4.5(26)	2.1 (12)	3.3(19)	7.9(45)
Stopping contraception	3.0(17)	0.7 (4)	4.9(28)	7.9(45)
Conception/fertility advice	3.8(22)	2.1 (12)	4.5(26)	6.5(37)
Other advice	0.9(5)	0.9 (5)	0.2(1)	1.2(7)
Given no advice and/or did not seek advice	45.7 (262)			

*Includes; finding out for themselves, Internet, books, TV/radio

The most frequent advice given to men in relation to specific preconception health behaviours was about eating a healthy diet (GP 6.8% n = 39, HP 2.8% n = 16, FF 19.3% n = 111); smoking (GP 7.3% n = 42, HP 1.9% n = 11, FF 12.7% n = 73,); alcohol (GP 7.2% n = 41, HP 2.4% n = 14, FF 14.7% n = 84,); caffeine (GP 4.5% n = 26, HP 1.7% n = 10, FF 7.8%, n = 45,) and BMI (GP 5% n = 29, HP 1.7% n = 10, FF 3.5% n = 20,). Table 2 details further behaviours.

19.1% of 565 men (n = 108) stated that they visited a GP or health professional to get advice about getting pregnant. 2.3% (n = 6) recalled being given preconception health advice for themselves only from GPs or health professionals. 24.5% (n = 63) recalled being given advice from GPs or health professionals for both themselves and their partner. 21% (n = 54) reported receiving information for their partner only.

### Behaviour change in preparation for pregnancy

Participants were asked about any actions they had taken to improve their health in preparation for the pregnancy. Over half (57.2%, n = 307) reported that they took no action to improve their health. Behaviours specifically relating to smoking, alcohol and taking vitamin supplements are presented in detail below. Other actions included eating more healthily (27.2% n = 146), seeking medical or health advice (5.8% n = 31) and taking other actions such as taking more exercise or drinking less caffeine (10.1% n = 54).

### Smoking behaviours

533 men answered questions about smoking behaviours. Almost half of these, 47.5% (n = 253) had never smoked and 52.5% (n = 280) had smoked at some point during their lives. 31.7% (n = 169) stated that they stopped smoking due to pregnancy and of these 78.7% (n = 133) stopped before conception although the exact interval before was not recorded. 15.8% of men (n = 84) stated they were still smoking when their partners were pregnant and of these 29. 8% (n = 25) were smoking more than 10 cigarettes a day. Few men stated that they smoked indoors at the time pregnancy was confirmed, 3.1% (n = 18 of 304 men), reducing to 0.7% (n = 4 of 304 men) at the time of questionnaire completion later in pregnancy.

### Alcohol behaviour

79.0% (n = 410) consumed alcohol in the 3 months before pregnancy. Of the 68 men (17%) who stopped drinking alcohol due to pregnancy, 67.6% (n = 46) stopped drinking before pregnancy and 32.4% (n = 22) gave up once their partner was pregnant. 72.4% (n = 389) were still drinking at the time of completing the questionnaire. 52.2% (n = 214) were drinking between 4–14 units per week and 22.7% (n = 93) over 14 units a week.

### Vitamins and supplements

Specific reasons for taking vitamins and supplements were not recorded but 28.4% (n = 163) of men reported to be taking them before their partner became pregnant. 68.1% (n = 111) were taking general multivitamins and minerals such as vitamin C, B12, Zinc and magnesium and 16.5% (n = 27) men were taking cod liver oil, fish oils or omega 3. 23.3% (n = 38) stated they took specific preconception vitamins for men, such as Wellman Conception or Pregnacare for men.

19.6% (n = 32) men reported to be taking a variety of dietary supplements including specific body building supplements and protein shakes, sports nutrition, powders and tablets.

### Factors influencing behaviour change

Having a planned pregnancy was an important factor influencing health behaviour change. Men who had a higher LMUP score, indicating a planned pregnancy, were significantly more likely to positively change their behaviour in reducing or stopping smoking, reducing alcohol and eating more healthily (Table 3).

**Table 3:** Characteristics of the sample by demographic and pregnancy intention for behaviour change.

Characteristic	% (n)	Reduced Smoking			Reduced Alcohol		Healthier Eating	
	All	No	Yes		No	Yes	No	Yes
**Age**								
**< = 25**	4.9 (19)	16	3		18	1	17	2
**26–30**	19.0 (78)	68	10		67	11	57	21
**31–35**	40.0 (164)	152	12		132	32	119	45
**36–40**	24.6 (101)	91	10		76	25	71	30
**41–45**	9.0 (37)	30	7		30	7	24	13
**> = 46**	2.4(10)	8	2		5	5	8	2
		*P = 0*.*269*			*P = 0*.*04*		*P = 0*.*499*	
**Ethnic group**								
**White**	72.2 (296)	265	31		228	68	221	75
**Mixed**	4.9 (20)	17	3		16	4	15	5
**Asian**	13.2 (54)	47	7		46	8	29	25
**Black**	6.1 (24)	22	2		23	1	20	4
**Chinese or Other**	3.7 (15)	14	1		15	0	11	4
		*P = 0*.*897*			*P = 0*.*038*		*P = 0*.*019*	
**General Health**								
**Excellent**	30.0 (122)	113	10		103	19	94	28
**Fair**	9.7 (40)	34	6		34	6	29	11
**Good**	60.0 (247)	219	28		191	56	173	74
		*P = 0*.*434*			*P = 0*.*189*		*P = 0*.*366*	
**BMI Group**								
**Normal**	49.5 (203)	178	25		162	41	146	57
**Overweight**	49.0 (200)	182	18		160	40	144	56
**Underweight**	1.5 (6)	5	1		6	0	6	0
		*P = 0*.*502*			*P = 0*.*471*		*P = 0*.*313*	
**Employment Status**								
**Employed or full-time education**	87.0 (356)	318	38		282	74	255	101
**Other**	2.6 (11)	9	2		10	1	10	1
**Part-time**	6.8 (28)	25	3		25	3	20	8
**Unemployed**	3.4 (14)	13	1		11	3	11	3
		*P = 0*.*844*			*P = 0*.*477*		*P = 0*.*518*	
**Highest Academic Qualifications**								
**A/AS/S levels**	5.1 (21)	18	3		18	3	14	7
**Degree**	68.7 (282)	260	22		222	60	203	79
**Diploma**	9.7 (40)	29	11		31	9	30	10
**Other**	16.3 (66)	58	8		57	9	49	17
		*P = 0*.*002*			*P = 0*.*466*		*P = 0*.*893*	
**Pregnancy Intention**								
**Unplanned**	1.2 (5)	5	0		5	0	5	0
**Ambivalent**	24.3 (99)	94	5		93	6	88	11
**Planned**	74.3 (305)	266	39		230	75	203	102
		*p<0*.*0001*			*p<0*.*0001*		*p<0*.*0001*	
**First Time Fathers**								
**Yes**	66.7 (273)	240	33		214	59	193	80
**No**	33.3 (136)	125	11		114	22	103	33
		*P = 0*.*29*			*P = 0*.*25*		*P = 0*.*34*	
**Complete cases (n = 409 No = No behaviour change reported**. **Yes = Behaviour Change reported (reduced smoking/alcohol. healthier eating)**.								

Being given or seeking preconception information was associated with increasing positive pre-pregnancy health behaviour. In relation to both smoking and diet (Table 4), information given by GPs or health professionals about smoking and pregnancy was associated with men being more likely to reduce smoking (Adjusted OR (95% CI) 3.24 (1.60–6.53) p = 0.001*). Similarly, information given by these professionals regarding eating a healthy diet was associated with eating more healthily (Adjusted OR (95% CI) 2.17 (1.20–3.92) p = 0.010*.

**Table 4:** Behaviour change for smoking, alcohol and dietary behaviour and information given by a health professional or any other source versus no information given.

Outcome	Level of information given & health professional input		
	No Information	Information given by GP/HP	Information given by any other source
**Reduced Smoking**			
**OR (95% CI)**	1-	3.70 (1.87–7.33) ***p<0*.*0001***	**2.66 (1.35–5.25)** ***p<0***.***0001***
**Adjusted^1^** **OR (95% CI)**	1-	3.24 (1.60–6.53) ***p = 0*.*001***	**2.21 (1.18–4.14)** ***p = 0***.***013***
**Reduced Alcohol**			
**OR (95% CI)**	1-	2.27 (1.26–4.09) ***p<0*.*006***	**5.55 (3.08–10.01)** ***p<0***.***0001***
**Adjusted^1^** **OR (95% CI)**	1-	1.62 (0.84–3.13) ***p = 0*.*150***	**4.68 (2.47–8.82)** ***p<0***.***0001***
**Healthier Eating**			
**OR (95% CI)**	1-	3.16 (1.83–5.46) ***p<0*.*0001***	**3.58 (2.24–5.73)** ***p<0***.***0001***
**Adjusted^1^** **OR (95% CI)**	**1-**	**2.17 (1.20–3.92)** ***p = 0***.***010***	**2.32 (1.44–3.72)** ***p = <0***.***0001***

^1^ Adjusted complete cases after multiple imputation. Adjusted for age, ethnicity, general health, academic qualification, employments status and LMUP score.

Being given or seeking information themselves from any other source also had a significant positive effect on promoting behaviour change in relation to reducing smoking (Adjusted OR (95% CI) (2.21 (1.18–4.14) p = 0.013*), eating more healthily (Adjusted OR (95%CI) 2.32 (1.44–3.72) p = <0.0001*) and reducing alcohol (Adjusted OR (95%CI) 4.68 (2.47–8.82) p<0.0001*. Data were adjusted for other factors associated with pre-pregnancy behaviour change including age, ethnicity, general health, academic qualifications, employment status and LMUP score.

Men who had obtained higher academic qualifications were significantly more likely to reduce or stop smoking *before* their partner’s pregnancy and reducing alcohol consumption was significantly associated with age and ethnicity (Table 3).

## Discussion

The health of men prior to conception is increasingly recognised as a factor for improving reproductive health, pregnancy and neonatal outcomes [6, 42]. Considerable concern has focused on paternal BMI, given the current obesity epidemic [3]. Preconception care allows optimisation of bio-psycho-social factors with potential to positively impact on men’s own health, that of their offspring and the experience of fatherhood [4].

Male spermatozoa contribute 50% of DNA at conception and recent studies indicate a global decline in quantity and quality of sperm [5, 7, 43].This appears to be due to a range of factors including lifestyle and environmental factors that cause DNA defragmentation through oxidative stress [44], resulting in poor sperm quality, and reduced successful pregnancy. Transgenerational studies also highlight the effect that nutrition and the environment can have on future generations through epigenetic processes. More recently research into the effect of social determinant policy has highlighted the influence on men’s health [45]. There is however a paucity of literature on men’s knowledge and behaviour in relation to PCC [46, 47] or of how to engage them in optimising their health prior to pregnancy.

In this study, attendance at their partner’s antenatal visit, coupled with the high level of pregnancy planning, and nearly a quarter taking preconception vitamins, implies that these men were relatively highly invested in pregnancy.

In this relatively well educated population, we found that almost half of men had made at least one positive behaviour change including eating more healthily, reducing smoking and reducing alcohol. This probably reflects their high levels of pregnancy planning. Changing behaviour was also related to being given information by GPs or other Healthcare Professionals or from other sources which they had actively sought.

Eating a healthier diet was the most common behaviour change, and giving up alcohol the least common with 79% drinking in the 3 months before pregnancy. We found that men who had a planned pregnancy and men who had received preconception information were both significantly more likely than other men to reduce smoking, reduce their alcohol consumption and to eat more healthily. These behaviours have the potential to improve men’s overall and sperm health subsequently leading to better maternal and neonatal health outcomes and positive fatherhood [4, 48–50].

### Planned pregnancy

As assessed by the LMUP, two thirds of men indicated that pregnancy with their partner had been planned. This correlates with the wider study of their female partners [33], which found that 73% of women had high LMUP scores indicating a planned pregnancy [36] and are consistent with figures from other UK studies in women [51, 52]. A recent Swedish study [46] which explored pregnancy planning behaviour in men using a Swedish Likert scale found 81% of pregnancies had been planned.

Women who have an unintended pregnancy have been found to have less optimal pregnancy health behaviours such as smoking and drinking alcohol, even after adjusting for socio-demographic characteristics [53, 54] and our findings are similar for men. Men who had a higher LMUP score indicating a planned pregnancy were significantly more likely to positively change lifestyle behaviours compared to those with lower LMUP scores.

Men’s role in planning pregnancy is nevertheless unclear. Studies which have explored attitudes, beliefs and behaviours of young males, have shown a discrepancy between their desire to prevent unintended pregnancy and their level of control with regard to this [55, 56]. Men may however influence partner decisions which may both directly and indirectly affect pregnancy. Misra et al. (2010) [57] discuss the role that paternal behaviours have in encouraging or discouraging maternal lifestyle choices and uptake of periconception care which may ultimately influence birth outcomes. Interventions such as the development of individual reproductive life-plans [23, 34] have been successful in increasing awareness of reproductive health issues with the aim of promoting planned pregnancy in both men and women.

### Preconception information

Receiving PCC information from health professionals or any source was associated with men improving their lifestyle behaviours. After adjusting for confounding factors associated with pre-pregnancy behaviour change including age, ethnicity, general health, academic qualification, employment status and LMUP score, this remained for reducing smoking and eating a healthier diet and for the small number of men who reduced alcohol before pregnancy, receiving or seeking information from any source other than Health Professionals was significantly associated with drinking less alcohol. This suggests that men may be more receptive to the promotion of PCC than previously recognised, given that they are usually portrayed as notoriously poor users of preventative health services and are less likely than women to visit their GP for preconception advice or interventions [58, 59]. The finding that if men receive information about PCC they reduced smoking and alcohol consumption should not be underestimated.

In our study men were more likely to reduce smoking than alcohol. Alcohol consumption was common with three quarters of the men drinking alcohol at the time of conception and nearly a quarter of these drinking over the UK government recommended 14 units of alcohol a week [60]. The influence of alcohol on sperm is equivocal with excessive amounts leading to poor quality sperm [61] but other studies disagreeing over moderate consumption [62]. Women are however advised to avoid alcohol around the time of conception and during pregnancy as no safe limit has been established and the effects of fetal alcohol spectrum disorders can be devastating [63, 64]. Nearly three quarters of men in our study continued to drink alcohol during pregnancy but other studies have found that men have given up drinking in support of their partners [46, 65].

Paternal smoking has a detrimental effect on neonatal outcomes. Men who smoke have altered semen qualities [66–68] and there is evidence for association between paternal smoking of cigarettes and conditions in offspring including cardiac malformations [69], cancers such as acute lymphoblastic leukaemia [70] and cleft lip palate anomalies [71]. Over half of our sample of men stated that they had been smokers and 32% had given up due to pregnancy with over three quarters quitting before conception. Nearly 15% continued smoking during the pregnancy and a third of these smoked more than 10 cigarettes a day. Recent studies examining effects of paternal smoking have found increased respiratory conditions including asthma in their offspring even when smoking has ceased before pregnancy [72]. There is therefore clear indication for education and PCC to target men who smoke to undertake quit smoking programmes.

Men in our study were more likely to seek information from other sources or to receive information about PCC from friends and family, rather than from GPs or Health Professionals. Only 2.3% received information specific to themselves from GPs or Health Professionals. Previous studies have found poor communication and little provision of PCC information by GPs and health professionals [24, 73], including a lack of interest from GPs in giving PCC to men unless subfertility is involved [47]. Increasing knowledge and awareness by training health professionals to communicate and deliver targeted PCC interventions may be important, but as men who received PCC information from any source were more likely to change behaviour, there is opportunity to develop innovative ways of promoting PCC. Evidence based Mobile Health interventions are being developed and tested which may encourage engagement with PCC [74–76].

### Need for preconception care

Studies from the USA [49, 59], Sweden [46] and Italy [77] have highlighted a substantial need for PCC amongst men. In the USA, Choiriyyah et al (2015) [59] using data from the National Survey of Family Growth, found that 60% of men aged 15–44 years were in need of PCC, based on their BMI, alcohol binge drinking and STI risk. Casey et al (2016) [49] using follow-up data from a National Survey of Adolescent males aged 35–39 years found that 33% had a need for PCC in relation to the age of partner, type of union and STI risk.

Despite a majority of men rating their own health as excellent or good, several factors indicate that in addition to their alcohol consumption and smoking habits, our study population also require PCC due to raised BMI, use of prescribed medications and low attendance for STI screening.

Nearly half of our sample was overweight or obese, characteristics that are negatively associated with male reproductive function. Men who have a BMI more than 30 are more likely to have hypogonadism with altered reproductive hormone profiles such as decreased testosterone and hyperestrogenisation impairing spermatogenesis [78]. Obesity is known to enhance oxidative stress disrupting sperm function through inflammatory processes in testicular tissue and semen [44]. Sperm quality is also of concern [13, 79, 80] and a systematic review and meta-analysis of 21 studies [81] found being overweight or obese was associated with an increased prevalence of azoospermia or oligozoospermia. Conversely studies have found that a low BMI less than 20 is also associated with poor sperm concentration in semen [78, 82]. Promotion of a healthy weight before conception is therefore an important part of PCC for men, further substantiated by recent transgenerational epigenetic studies indicating paternal obesity can reprogramme metabolic and reproductive stem cells leading to adiposity in their offspring [83].

Almost a third of men taking medications in our study were taking medication that had potential for adverse effect on reproductive health either through genetic influence such as aneuploidy or poor sperm quality [84] or through affecting reproductive function. Although half had attended for medication review in the past three months, it is unclear if medication was discussed in relation to pregnancy. In a Norwegian study 25% of fathers had been prescribed medication in the three months prior to conception [32] and men have been found to be unaware of the effect on spermatogenesis, sperm production and erectile function [85]. Common prescription medication such as anti-depressants, antibiotics, antifungals and recreational drug use such as marijuana, cocaine, anabolic steroids and hair loss products affect sperm quality [6, 85]. In our study 19.6% of the 28.4% men who were taking vitamins and supplements, stated taking body building supplements which may contain steroids. Environmental and occupational hazards may also need consideration as exposure to chemicals, heavy metals and radiation can affect sperm quality and the ability to conceive [6, 86].

Sexually transmitted infections such as gonorrhoea, chlamydia, syphilis and human immunodeficiency virus (HIV) can affect fertility, pregnancy and cause congenital malformations [87, 88] and although we did not specifically assess STI risk, only 13.6% had attended for STI screening prior to conception. STIs may be asymptomatic and screening and treatment of infection prior to pregnancy may be appropriate [6].

### Strengths and limitations

Strengths of this study are found in the high response rate and successful recruitment of fathers as they attended early pregnancy scan clinics with their partners. However, we recognise that our sample may not be representative in that the men had a high level of education (67% had a degree) and 87% were employed or were in full time education. This may have introduced bias in that we recruited men who could afford to take time off work to attend scans with their partner. Despite the three hospitals being chosen for their diverse populations, 74% of our participants described their ethnicity as White.

Questions about the pre-pregnancy period are necessarily retrospective and recall may have introduced bias.

### Implications for future research policy and practice

Currently most PCC is focused on women, but in light of robust evidence that factors such as smoking, drugs and alcohol affect sperm health, and that men are more likely to engage in risk behaviour, there is growing interest in evaluating interventions for men [46, 89]. In the USA Center for Disease Control (CDC) programmes, men are recognised as fundamentally important to the health and wellbeing of the next generation and are an integral target for preconception care strategies and policy [4, 90]. In Europe, there are few guidelines in regards to preconception care for men [91] and although there is recognition of the importance of men’s health on reproductive health, research has yet to influence policy.

Men are less likely to access healthcare [52] and therefore our finding that PCC information from any source had an effect on behaviour change is important. There is a clear need for evaluation of innovative public health interventions that engage men from all socio-economic groups through everyday life activities as well as via health care.

Studies are emerging that use digital health interventions to promote behaviour change [92] and in the UK guidelines are being developed for use of technology-based interventions [93], which help people achieve health goals such as managing weight, quitting smoking or reducing alcohol, all of which are all relevant to preconception care. Men have also been found to positively influence their partners lifestyle decisions in respect of pregnancy [94, 95] and further research should consider health behaviour interventions which target couples.

### Conclusion

To our knowledge, this is the first study in the UK to examine preconception care in men. Our findings that men who had planned pregnancy and men that had received preconception information were more likely than other men to reduce smoking, reduce alcohol consumption and to eat more healthily has potential to optimise paternal reproductive health, maternal health and to improve pregnancy and neonatal outcomes.

Previous studies with similarly high levels of pregnancy planning, have presented more negative findings about men’s awareness of preconception health and propensity for preconception behaviour change. By contrast we found that over two fifths of the men, who were sufficiently invested in a pregnancy to accompany their partners to hospital, had good levels of awareness as indicated by their making at least one positive health behaviour change in reducing smoking, alcohol or improving their diet.

However, in objective terms, the men in our study were not particularly healthy with nearly half being overweight or obese, which together with their alcohol consumption and smoking habits indicates the challenge of improving men’s health before conception.

Our study highlights that preconception care information given by GPs or Health Professionals or sought from other sources has the potential to increase positive pre-pregnancy health behaviour in men. Greater attention needs be given to maximise this impact by developing innovative approaches to promoting PCC information and positive fatherhood to all men.

## Supporting information

S1 File Antenatal partner data file_2019 full. The file contains the anonymised raw data used in this manuscript.
(CSV)
